# Neuroprotective Herbs for the Management of Alzheimer’s Disease

**DOI:** 10.3390/biom11040543

**Published:** 2021-04-08

**Authors:** Julie Gregory, Yasaswi V. Vengalasetti, Dale E. Bredesen, Rammohan V. Rao

**Affiliations:** 1 Apollo Health, P.O. Box 117040, Burlingame, CA 94011, USA; julie@ahnphealth.com; 2 College of Medicine, University of Central Florida, Orlando, FL 32827, USA; yasaswi123@gmail.com; 3 Department of Molecular and Medical Pharmacology, University of California, Los Angeles, CA 90024, USA; 4 California College of Ayurveda, 700 Zion Street, Nevada City, CA 95959, USA

**Keywords:** herbs, Alzheimer’s disease, neurodegeneration, ashwagandha, brahmi, cat’s claw, ginkgo biloba, gotu kola, lion’s mane, saffron, shankhpushpi, turmeric, triphala

## Abstract

Background—Alzheimer’s disease (AD) is a multifactorial, progressive, neurodegenerative disease that is characterized by memory loss, personality changes, and a decline in cognitive function. While the exact cause of AD is still unclear, recent studies point to lifestyle, diet, environmental, and genetic factors as contributors to disease progression. The pharmaceutical approaches developed to date do not alter disease progression. More than two hundred promising drug candidates have failed clinical trials in the past decade, suggesting that the disease and its causes may be highly complex. Medicinal plants and herbal remedies are now gaining more interest as complementary and alternative interventions and are a valuable source for developing drug candidates for AD. Indeed, several scientific studies have described the use of various medicinal plants and their principal phytochemicals for the treatment of AD. This article reviews a subset of herbs for their anti-inflammatory, antioxidant, and cognitive-enhancing effects. Methods—This article systematically reviews recent studies that have investigated the role of neuroprotective herbs and their bioactive compounds for dementia associated with Alzheimer’s disease and pre-Alzheimer’s disease. PubMed Central, Scopus, and Google Scholar databases of articles were collected, and abstracts were reviewed for relevance to the subject matter. Conclusions—Medicinal plants have great potential as part of an overall program in the prevention and treatment of cognitive decline associated with AD. It is hoped that these medicinal plants can be used in drug discovery programs for identifying safe and efficacious small molecules for AD.

## 1. Introduction

Alzheimer’s disease (AD) is one of the most significant global healthcare problems and is now the third leading cause of death in the United States [1,2,3]. While the etiology is incompletely understood, genetic factors account for the 5 to 10% of cases that are familial Alzheimer’s, with the other 90 to 95% being sporadic. Being heterozygous or homozygous for the ApoE ε4 allele significantly increases the risk of developing Alzheimer’s. Efforts to find a cure for AD have so far been disappointing, and the drugs currently available to treat the disease have limited effectiveness, especially if the disease is in its moderate–severe stage.

The underlying pathology is neuronal degeneration and loss of synapses in the hippocampus, cortex, and subcortical structures. This loss results in gross atrophy of the affected regions, resulting in loss of memory, inability to learn new information, mood swings, executive dysfunction, and an inability to complete activities of daily living (ADLs). Patients in the late–severe stage of AD will require comprehensive care owing to complete loss of memory and the disappearance of their sense of time and place. It is believed that therapeutic intervention that could postpone the onset or progression of AD would dramatically reduce the number of cases over the next 50 years [1,2].

The two prominent pathologic hallmarks of Alzheimer’s disease are (a) extracellular accumulation of β-amyloid deposits and (b) intracellular neurofibrillary tangles (NFT). Accumulated Aβ triggers neurodegeneration, resulting in clinical dementia that is characteristic of AD [4,5,6]. However, the poor correlation of amyloid deposits with cognitive decline in the symptomatic phase of dementia may explain why drug targets to β-amyloid have not succeeded to date [5,6].

Intracellular neurofibrillary tangles (NFTs) are commonly seen in AD brains and represent aberrantly folded and hyperphosphorylated isoforms of the microtubule-associated protein tau [7,8]. Studies reveal that the mutated, aberrantly folded, and hyperphosphorylated tau is less efficient in sustaining microtubule growth and function, resulting in the destabilization of the microtubule network—a hallmark of AD [9]. Attention is now on therapies targeted at tau due to failures in β-amyloid clinical drug trials [7,8,10]. However, the recent failure of drugs targeting tau deposits suggests a lack of accurate understanding of the complex pathophysiology of AD [11]. This demonstrates the need to consider other pathophysiological entities underlying AD, including, but not limited to, autophagy, neuroinflammation, oxidative stress, metal ion toxicity, neurotransmitter excitotoxicity, gut dysbiosis, unfolded protein response, cholesterol metabolism, insulin/glucose dysregulation, and infections [12]. In the face of repeated failures of drug therapies targeting amyloid or tau and the large unmet need for safe and effective AD treatments, it is imperative to pursue alternative therapeutic strategies that address all the above-mentioned pathophysiological entities [13,14].

We reported the first examples of reversal of cognitive decline in AD and pre-AD conditions including mild cognitive impairment (MCI) and subjective cognitive impairment (SCI), using a comprehensive, individualized approach that involves determining the potential contributors to the cognitive decline. Some examples of addressing these potential contributors include: (1) identifying gastrointestinal hyperpermeability, repairing the gut, and optimizing the microbiome; (2) identifying insulin resistance and returning insulin sensitivity; (3) reducing protein glycation; (4) identifying and correcting suboptimal levels of nutrients, hormones, and trophic molecules; (5) identifying and treating pathogens such as Borrelia, Babesia, or Herpes family viruses; and (6) identifying and reducing levels of metallotoxins, organic toxins, or biotoxins through detoxification procedures. This sustained effect of the personalized, precision therapeutic program represents an advantage over monotherapeutics [15]. Included in this individualized, precision program are high-quality herbs or their bioactive compounds directed towards the specific needs of each patient as part of the overall protocol, and these have proven to be very effective.

While herbs and herbal remedies have a long history of traditional use and appear to be safe and effective, they have unfortunately received little scientific attention [16,17,18,19,20]. Numerous plants and their constituents are recommended in traditional practices of medicine to enhance cognitive function and to alleviate other symptoms of AD, including poor cognition, memory loss, and depression. A single herb or a mixture of herbs is normally recommended depending upon the complexity of the condition. The rationale is that the bioactive principles present in the herb not only act synergistically but may also modulate the activity of other constituents from the same plant or other plant species [20,21,22]. This approach has been used in Ayurveda, traditional Chinese medicine (TCM), and Native Americans’ system of medicine, where a single herb or a combination of two or more herbs is commonly prescribed for any specific disease [16,17,18,19,23]. In this manuscript, we review a subset of herbs useful for AD based on their properties, functional characteristics, and mechanistic actions (Table 1). The rationale for choosing these herbs is (a) their long historical use in traditional practices of medicine for memory-related disorders including AD, (b) the identification of phytochemicals from these plant sources for their potential in AD therapy, (c) determination of the neuropharmacological activities of these herbs, and (d) pre-clinical or clinical studies to confirm their reputed cognitive-enhancing and anti-dementia effects.

It is hoped that the historical knowledge base of traditional systems of medicine, coupled with combinatorial sciences and high-throughput screening techniques, will improve the ease with which herbal products and formulations can be used in the drug development process to provide new functional leads for AD.

### 1.1. Ashwagandha (Withania somnifera)

Ashwagandha, commonly called Indian ginseng or winter cherry, is one of the most prominent herbs prescribed as a brain rejuvenator for AD. It is prescribed to increase energy, improve overall health and longevity, and as a nerve tonic [86]. Ashwagandha has been shown to possess antioxidant activity, free radical scavenging activity, as well as an ability to support a healthy immune system [87]. Ashwagandha contains several bioactive compounds of great interest, such as ergostane-type steroidal lactones, including withanolides A-Y, dehydrowithanolide-R, withasomniferin-A, withasomidienone, withasomniferols A-C, withaferin A, withanone, and others. Other constituents include the phytosterols sitoindosides VII-X and beta-sitosterol and alkaloids [86,88].

A subset of these components has been shown to scavenge free radicals generated during the initiation and progression of AD. Molecular modeling studies showed that withanamides A and C uniquely bind to the active motif of Aβ25-35 and prevent fibril formation. Furthermore, these compounds protected PC-12 cells and rat neuronal cells from β-amyloid-induced cell death [89,90,91]. Treatment with the methanol extract of ashwagandha triggered neurite outgrowth in a dose- and time-dependent manner in human neuroblastoma cells [29], and, in another study involving cultured rat cortical neurons, treatment with Aβ peptide induced axonal and dendritic atrophy and loss of pre-and postsynaptic stimuli [92]. Subsequent treatment with withanolide A induced significant regeneration of both axons and dendrites and restored the pre- and post-synapses in the cultured cortical neurons.

In vivo, withanolide A inhibited Aβ(25–35)-induced degeneration of axons, dendrites, and synapses in the cerebral cortex and hippocampus and also restored Aβ-peptide-induced memory deficits in mice [93]. The in vivo ameliorative effects were maintained even after the discontinuation of the drug administration. Aqueous extracts of ashwagandha increased acetylcholine (ACh) content and choline acetyl transferase activity in rats, which might partly explain the cognition-enhancing and memory-improving effects [29,94,95]. Treatment with the root extract caused the upregulation of the low-density lipoprotein receptor-related protein, which enhanced the Aβ clearance and reversed the AD pathology in middle-aged and old APP/PS1 mice [96].

Oral administration of a semi-purified extract of ashwagandha reversed behavioral deficits and blocked the accumulation of Aβ peptides in an APP/PS1 mouse model of AD. This therapeutic effect of ashwagandha was mediated by the liver low-density lipoprotein receptor-related protein [96]. Using an AD model of Drosophila melanogaster, researchers noted that treatment with ashwagandha mitigated Aβ toxicity and also promoted longevity [97]. Despite the extensive literature on the therapeutic effects of ashwagandha, there are limited data on its clinical use for cognitive impairment [98].

In a prospective, randomized, double-blind, placebo-controlled pilot study involving 50 subjects with mild cognitive impairment, subjects were treated with either ashwagandha root extract (300 mg twice daily) or placebo for eight weeks. After eight weeks of study, the ashwagandha treatment group demonstrated significant improvements in both immediate and general memory tests compared to the placebo group. Furthermore, the treatment group showed significant improvement in executive function, sustained attention, and information-processing speed [99]. These studies lend credence to ashwagandha’s role in enhancing memory and improving executive function in people with SCI or MCI.

### 1.2. Brahmi (Bacopa monnieri)

Brahmi, or *Bacopa monnieri* (Bm), is a perennial creeper medicinal plant found in the damp and marshy wetlands of Southern and Eastern India, Australia, Europe, Africa, Asia, and North and South America. In the Ayurvedic system of medicine, Bm is recommended for mental stress, memory loss, epilepsy, insomnia, and asthma [34,36]. The bioactive phytochemicals present in this plant include saponins, bacopasides III, IV, V, bacosides A and B, bacosaponins A, B, C, D, E, and F, alkaloids, sterols, betulic acid, polyphenols, and sulfhydryl compounds, which may be responsible for the neuroprotective roles of the plant. Both in vitro and in vivo studies show that these phytochemicals have an antioxidant and free radical scavenging action by blocking lipid peroxidation in several areas of the brain [36,100,101,102]. Bm acts by reducing divalent metals, scavenging reactive oxygen species, decreasing the formation of lipid peroxides, and inhibiting lipoxygenase activity [103].

Numerous studies have also shown Bm’s role in memory and intellect [33,56,100,104,105,106]. To determine the neuroprotective effect of Bm in a rat model of AD, researchers tested an alcoholic extract of Bm at doses of 20, 40, and 80 mg/kg for a period of 2 weeks before and 1 week after the intracerebroventricular (icv) administration of ethylcholine aziridinium ion (AF64A). Spatial memory was tested using the Morris water maze (MWM), and the cholinergic neuron density was determined using histological techniques. The researchers showed that Bm extract improved the escape latency time in the MWM test and blocked the reduction of cholinergic neuron densities [35]. Another group reported the reversal of colchicine-induced cognitive deficits by a standardized extract of Bm. In addition to reversing colchicine-triggered cognitive impairment, the Bm extract also attenuated colchicine-induced oxidative damage by decreasing the protein carbonyl levels and restoring the activities of the antioxidant enzymes [107].

Most of the studies exploring the cognitive-enhancing effects of Bm in humans focused on normal, aged individuals. In a double-blind, randomized, placebo-controlled trial on 35 individuals aged above 55 years, subjects received either 125 mg of Bm extract or a placebo twice a day for a period of 12 weeks, followed by a placebo period of another four weeks. Subjects underwent a battery of memory tests, including general information, orientation, mental control, logical memory, digit forward, digit backward, visual reproduction, and paired association learning. Subjects were scored on each sub-test, and total memory score was calculated by adding the score of all subtests. A significant improvement was observed in mental control, logical memory, and paired association learning in Bm-treated patients compared to the placebo group at 8 and 12 weeks after initiation of the trial [37]. The results suggested the use of Bm in the treatment of age-associated memory impairment.

Ten subjects were given 500 mg of Sideritis extract, 320 mg Bm extract, or a combination using a crossover design. Sideritis extract is rich in a variety of flavonoids and has been shown to improve cognition in animal models of AD [108]. The Attention d2 Test is a neuropsychological measure of selective and sustained attention and visual scanning speed. Assessment tests revealed that Sideritis extract combined with a low-dose Bm extract resulted in improvement in the d2 concentration test score [109]. A similar effect of Bm alone was observed only after repetitive dosing, suggesting that the long-term memory effects seen with repetitive dosing of Bm may be a promising therapeutic option for subjects suffering from MCI [109].

In another prospective, non-comparative, multicenter trial involving 104 subjects who suffered from MCI, Bm extract in combination with astaxanthin, phosphatidylserine, and vitamin E was given for 60 days. The tested combination formula was well tolerated. Cognitive and mnemonic performance was assessed with validated instruments including Alzheimer’s Disease Assessment Scale-Cognitive Subscale (ADAS-cog) and Clock-Drawing Test (CDT) that can assess the risk of MCI progression to AD. Researchers noted significant improvements in ADAS-cog and CDT scores [110]. The observed sixty-day improvements in ADAS-cog and CDT were statistically significant as compared with baseline values. Memory is affected by several factors, including focus and attention, neurotransmitters, hormones, trophic factors, cyclic AMP, ion channels, protein transcription, synapse formation, and nutrients. Some of these processes can be modulated by Bm extract alone or in combination with other compounds.

The abovementioned study design is similar to our therapeutic program for people with SCI and MCI, where Bm is administered in combination with other nutraceuticals and cogniceuticals [15,111].

### 1.3. Cat’s Claw (Uncaria tomentosa)

Cat’s claw (CC) is a tropical vine with hooked thorns that resemble the claws of a cat and is mainly recommended for its potential role in the treatment of AD and pre-AD. It is found mainly in the Amazon rainforest and other areas of South and Central America. This medicinal plant contains oxindole alkaloids, polyphenols (flavonoids, proanthocyanidins, and tannins), glycosides, pentacyclic alkaloids, and sterols [38,39]. CC is known for its immune-modulating and anti-inflammatory effects and for its role as a free radical scavenger. Based on in vitro studies, the anti-inflammatory effect of CC is attributed to its ability to inhibit iNOS gene expression, nitrate formation, cell death, PGE2 production, and the activation of NF-κB and TNF-α [45].

Using a transgenic mouse model of Alzheimer’s disease, a significant reduction in the Aβ load (by 59%) and plaque number (by 78%) in the hippocampus and cortex was observed after treating 8-month-old mice with the CC extract for 14 days [44]. CC extract also caused a significant reduction in astrocytosis and microgliosis, and it improved hippocampus-dependent memory. Some of the components in the CC extract crossed the blood–brain barrier (BBB) and entered the brain parenchyma following intravenous injection [44].

Pre-clinical studies suggest that CC extract inhibits the formation of plaques and tangles, reduces astrocytosis and microgliosis and improves memory in mouse models of AD [43,44]. CC extract not only prevented the formation and aggregation of Aβ fibrils and tau protein paired helical filaments, but it also facilitated the disaggregation of preformed fibrils and tau protein tangles [43,44]. While proanthocyanidin B2 was identified as the primary phytochemical with plaque-and tangle-dissolving activity, other polyphenols present in the CC extract also possess plaque-reducing activity [44].

Based on pre-clinical studies, Cat’s claw may be effective for memory loss and cognitive decline associated with AD, although no studies have been carried out in humans.

### 1.4. Ginkgo Biloba

Ginkgo biloba (Gb) has been in the spotlight primarily for its potential role in treating AD. Gb also appears promising as a therapeutic agent for several other chronic and acute forms of diseases. The main pharmacologically active groups of compounds are flavonoids and terpenoids. Almost all clinical studies use Gb extract that contains a combination of flavonoid glycosides, terpene lactones, and ginkgolic acids [50]. Gb extract has shown beneficial effects in treating Alzheimer’s, cardiovascular diseases, cancer, tinnitus, and other age-associated conditions [49,50]. The suggested mechanisms of the Gb extract are its antioxidant effect, anti-platelet activating factor activity for vascular diseases, inhibition of β-amyloid peptide aggregation in AD, and decreased expression of peripheral benzodiazepine receptor for stress alleviation [48,49,50].

Gb is popular as a treatment for early-stage AD and vascular dementia. Gb extract reverses β-amyloid and NO-induced toxicity in vitro and reduces apoptosis both in vitro and in vivo [112,113,114]. Treatment with Gb extract enhanced memory retention in young and old rats and improved short-term memory in mice [49,115].

Several studies indicate that ginkgo delays the progression of AD and is as effective as the cholinesterase inhibitors for treating AD. A modest improvement in cognitive function was observed in AD subjects in various randomized, double-blind, placebo-controlled trials [116,117,118]. Gb extract also improves ADLs among AD individuals and is preferred over other AD medications because of its negligible adverse effects [119,120].

### 1.5. Gotu Kola (Centella asiatica)

Considered both a nutraceutical and cogniceutical, Gotu kola (Gk) is a staple in Chinese, Indonesian, and Ayurvedic medicine [57]. This medicinal plant is used to strengthen the brain, heal skin issues, and promote liver and kidney health. Gk is considered a rejuvenating herb for nerve and brain cells as it is believed to promote intelligence and improve memory [54,55,56,57]. In vitro studies using various Gk plant derivatives (asiaticosides, asiatic acid, madecassoside, and madasiatic acid) showed that these compounds were capable of blocking H_2_O_2_-induced cell death, decreasing free radical concentration, and inhibiting β-amyloid cell death, suggesting a potential role for Gk in the treatment and prevention of Alzheimer’s disease [55,58,121,122].

Gk ethanolic extract triggered neurite outgrowth in human SH-SY5Y cells in the presence of nerve growth factor (NGF) and accelerated axonal regeneration in rats. Gk leaf extract showed improvement in learning and memory in rats by modulating several neurotransmitters, including dopamine, 5-hydroxytryptamine, and noradrenaline, in the rat brain, suggesting a potential therapeutic role in the treatment of AD-associated cognitive change [123,124].

Using PSAPP transgenic mice that spontaneously develop Aβ plaques, researchers observed that treatment with 2.5 mg/kg of Gk extract significantly decreased Aβ1–40 and Aβ1–42 levels in the hippocampus. Furthermore, long-term treatment with a higher dose of Gk aqueous extract resulted in a significant reduction in Congo red positive fibrillar amyloid plaques. Significant reactive oxygen species scavenging activity was detected with the lowest dose of Gk extract. Gk also significantly inhibited H_2_O_2_-induced lipid peroxidation and DNA damage [125].

Several derivatives of asiatic acid, one of the phytochemicals present in Gk, showed significant cognitive-enhancing activity in a scopolamine-induced memory impairment model. Scopolamine induces transient memory deficits similar to early AD. Using passive avoidance and Morris water maze tests, researchers observed that pre-treatment with three different derivatives of asiatic acid significantly improved memory compared to scopolamine-treated mice that were not given any drugs. The cognition-enhancing effect of these derivatives was due to increased choline acetyltransferase activity, resulting in improved ACh synthesis [126].

In a randomized, placebo-controlled, double-blind study, Gk extract was administered to 28 healthy volunteers at various doses (250, 500, and 750 mg) once daily for two months. Cognitive performance and mood were assessed prior to the trial, after the first administration, and one and two months after treatment. The results showed that the high dose of the plant extract enhanced working memory. Improvements were also noted in self-rated mood following the Gk treatment, suggesting the potential of Gk in mitigating age-associated decline in cognitive function and mood swings in the healthy elderly [52].

### 1.6. Lion’s Mane (Hericium erinaceus)

Lion’s mane (Lm) is an edible mushroom that is predominant in North America, Europe, and Asia. It is widely used in traditional Chinese medicine for its neuroprotective, anti-cancer, and anti-inflammatory properties [59]. These benefits are attributed to the two principal constituents of Lm, namely hericenones and erinacines [127,128]. Using several cell lines, researchers observed that treatment with Lm extract increased the expression of nerve growth factor (NGF) [127,129]. Lm extract was able to stimulate neurite length in the presence of NGF both in cell lines and cultured neurons [62].

Increased hippocampal neurogenesis and improvement in cognitive performance were observed in aged mice that were fed Lm extract for two months. Treatment of AD mice with Lm extract resulted in the reduction of Aβ plaques and elevation of NGF levels. Additionally, Lm extract also improved behavior, increased expression of insulin degrading enzyme, increased neurogenesis, and reduced astro- and microgliosis [61,62,63]. Lm extract also also improved cognition and increased the levels of acetylcholine and choline acetyltransferase in a mouse model of AD [130].

In a double-blind, parallel-group, placebo-controlled study involving 30 patients with MCI, a 16-week treatment with 3000 mg of Lm extract resulted in increased scores on the cognitive function scale in the experimental group compared to the placebo group [131]. In another small, randomized study involving patients with mild AD, Lm extract improved scores on the activities of daily living (e.g., personal hygiene, dressing, preparing food, etc.) over 49 weeks [132]. Thus, the abovementioned pre-clinical and clinical results suggest that lion’s mane is a well-tolerated and safe herb for the management of AD.

### 1.7. Saffron (Crocus sativus)

Saffron is a crimson-colored spice that is widely cultivated in Iran, India, and Greece. In addition to its usage in the textile and cosmetic industries, saffron is also recommended for its medicinal properties [65,66]. The major component of saffron is safranal, a carboxaldehyde. In vitro and in vivo studies show that the phytochemicals present in saffron possess antioxidant, anti-inflammatory, and anti-amyloidogenic properties [64,65,66].

To assess the efficacy of saffron in the treatment of mild to moderate AD, researchers enrolled forty-six patients that were randomly assigned to receive saffron 30 mg/day or placebo. After sixteen weeks, saffron produced a significantly better outcome on cognitive performance (ADAS-cog and CDR scores) than placebo. The double-blind, placebo-controlled study suggested that saffron was safe and effective in mild to moderate AD [133].

In an extension of the above study, researchers compared saffron extract with the cholinesterase inhibitor donepezil in subjects with mild to moderate AD. In a twenty-two-week double-blind, randomized, controlled trial, 54 participants were randomly administered either a 30 mg/day capsule of saffron or 10 mg/day of donepezil. At the end of the study, saffron had a similar effect in the improvement of cognitive function in subjects with AD as donepezil but with fewer side effects compared to donepezil. The researchers noted that saffron’s ability in treating mild-to-moderate AD might be due to its ability to inhibit the aggregation and deposition of beta-amyloid plaques [134].

A safety and efficacy pilot study was conducted by comparing saffron extract with memantine in reducing cognitive defects. Sixty-eight patients with moderate to severe AD were enrolled in a randomized, double-blind, parallel-group study. Subjects received memantine (20 mg/day) or saffron extract (30 mg/day) capsules for twelve months. In addition to showing a low rate of adverse effects, the saffron extract was also comparable with memantine in reducing cognitive decline in patients with moderate to severe AD [135].

Thus, all of the above-mentioned studies have found saffron to be an herbal spice with the potential to improve cognitive function and ADLs in patients with AD and MCI. While saffron possesses the ability to treat patients with AD as effectively as conventional treatment, it is a safer alternative because it is natural and has fewer adverse effects.

### 1.8. Shankhpushpi (Convolvulus pluricaulis)

Shankhpushpi, or *Convolvulus pluricaulis* (Cp), is used for nerve regeneration and for improvement of memory [68,70,91,136]. The major chemical components include triterpenoids, flavonol glycosides, anthocyanins, and steroids, which are responsible for Cp’s nootropic and memory-enhancing properties [67,68,69,137]. Cholinergic and glutamatergic signaling can be enhanced by a group of nutraceuticals called racetams. Cp produces some similar effects to racetams. Cp modulates the body’s production of adrenaline and cortisol [69]. Cp is also recommended for mental stress and fatigue, anxiety, and insomnia [36,68,106].

An ethanolic extract of Cp displayed significant antioxidant activity when tested in vitro and significantly improved learning and memory in rats [91,136,138,139]. Administration of aqueous root extract of Cp to neonatal rat pups resulted in improved retention and spatial learning performance. In addition, a significant increase in ACh content and activity was observed, which may be the basis for their improved learning and memory [140,141,142]. A significant increase in dendritic branching points and processes was observed in rats treated with Cp extract as compared to age-matched saline controls, suggesting that Cp improves learning and memory by stimulating dendritic arborization [143].

Similarly, administration of Cp extract showed a dose-dependent increase in acetylcholine esterase activity in CA1 and CA3 regions associated with the learning and memory functions, both in young and old mice, although memory retention was better in young mice [144]. Despite the vast literature demonstrating the in vitro and in vivo therapeutic properties of Cp, the herb has not been evaluated clinically to test whether it can prevent dementia.

### 1.9. Turmeric (Curcuma longa)

Turmeric is a flowering plant of the ginger family Zingiberaceae and is native to the Indian subcontinent and Southeast Asia. The bright yellow–orange color that this rhizome plant displays is mainly due to the polyphenolic compounds called curcuminoids. Turmeric is anti-inflammatory, antiseptic, and antibacterial and has long been used to treat a wide variety of conditions including liver detoxification, to prevent infection and inflammation, to balance cholesterol levels, to treat allergies, to stimulate digestion, and to boost immunity [80]. The active constituents of turmeric are turmerone oil and water-soluble curcuminoids. Curcuminoids include curcumin, demethoxycurcumin (DMC), bisdemethoxycurcumin (BDMC), and cyclocurcumin [81]. Curcumin is the principal curcuminoid whose anti-inflammatory property is associated with reduced risk of AD [82]. In vitro studies revealed curcumin’s ability to block lipid peroxidation and neutralize reactive oxygen species, which was several times more potent than vitamin E [145].

Oral administration of curcumin to aged mice with advanced plaque deposits resulted in a significant reduction in the plaque load [77,78,146,147]. Curcumin also reduced inflammation, oxidative damage, and amyloid pathology in mouse models of AD [78,147]. Direct injection of curcumin into the brains not only blocked further development of plaque but also reduced the plaque levels [147]. Using animal models of AD, several studies have reported improvement in cognitive function in the curcumin-treated group. Researchers attribute the improvement to curcumin’s ability to lower Aβ plaque levels as well as to its anti-inflammatory and antioxidant properties [148,149,150]. Using an APP/PS1 double transgenic AD model, researchers examined the effect of two different doses of curcumin, including low (160 ppm) and high (1000 ppm), after administration for six months in the diet [151]. While there was a significant cognitive improvement at both doses compared to the untreated group, the higher dose of curcumin produced greater cognitive improvement. Additionally, curcumin reduced the Aβ deposits, possibly by promoting autophagy. Owing to curcumin’s low bioavailability, rapid gastrointestinal metabolism, and poor BBB penetration, several analogs of curcumin were tested for their bioavailability and for their effects on animal models of AD. While these derivatives produced different beneficial outcomes depending on the disease model, they were all better in improving cognitive function and reducing plaque pathology [85].

Curcumin also reverses cognitive impairments in various animal models of AD. Higher doses of curcumin are more effective compared to the lower doses regardless of the route of administration, and improvements in cognition were greater when curcumin was given in combination with piperine, which has numerous pharmacological effects and several health benefits, especially against chronic diseases [84]. Metals such as copper, zinc, or iron may play a role in AD pathogenesis [152]. These metals are concentrated in the AD brain and trigger amyloid aggregation or oxidative neurotoxicity [153]. Curcumin forms strong complexes with metals and blocks metal-triggered Aβ aggregation, toxicity, and inflammation [154,155].

Contrary to animal studies, only a limited number of clinical studies have examined curcumin’s effect on human cognitive functioning, and the results are inconclusive. Researchers are nearly unanimous in their opinion that a combination of curcumin with other dietary supplements, such as piperine, α-lipoic acid, *N*-acetylcysteine, B vitamins, vitamin C, and folate, has a synergistic effect and enhances its neuroprotective effects [83,84,85]. Thus, improvements are needed, and future research should focus on ways to further increase curcumin’s systemic bioavailability and improve its BBB permeability.

### 1.10. Triphala

Triphala (Tri = three, phala = fruits) is a combination of three fruits or three myrobalans, namely Amalaki (*Emblica officinalis*; *Phyllanthus emblica*), Bibhitaki (*Terminalia bellerica*), and Haritaki (*Terminalia chebula*). They are usually mixed at a 1:1:1 ratio. Triphala is the therapeutic herb of choice for the treatment of several metabolic diseases, dental issues, skin conditions, eye diseases, heart conditions, hypercholesterolemia, colon issues, gingivitis, dental cavities, and treatment of cancer [71,72]. Research studies with triphala show that it may have antioxidant, anti-inflammatory, immunomodulatory, antibacterial, antimutagenic, hypoglycemic, and antineoplastic effects [71,73]. In vitro studies suggest that triphala is a modulator of cytochrome P450 and combats degenerative and metabolic disorders, possibly by inhibiting lipid peroxide formation and scavenging free radicals [74]. All three fruits possess different bioactive compounds with different properties.

Amalaki is a rich source of vitamin C and also contains phenols, tannins, and other compounds that have anti-cancer properties [71,72,73]. Furthermore, amalaki suppresses neurodegeneration in fly models of Huntington’s and Alzheimer’s diseases, thereby revealing its broad therapeutic potential [156,157]. Bibhitaki contains tannins, ellagic acid, gallic acid, lignans, and flavones, which have anti-inflammatory and antidiabetic properties [71,158]. Haritaki contains terpenes, polyphenols, anthocyanins, and flavonoids and is thought to have anti-inflammatory, anti-bacterial, anti-viral, and antioxidant properties while also improving digestive disturbances [71,158]. Other bioactive compounds present in triphala include gallic acid, tannins, chebulinic acid, ellagic acid, quercetin, luteolin, and saponins, all of which have antioxidant properties [159]. Triphala-derived polyphenols such as chebulinic acid are transformed by the human microbiota into bioactive metabolites such as urolithins [160,161]. Urolithins induce cellular autophagy and increase lifespan and inhibit muscle dysfunction in animal models of aging [162].

In mice fed a high-fat diet, supplementation with triphala resulted in significant reductions in body weight, waist circumference, hip circumference, energy intake, and percentage of body fat. Triphala lowered serum total cholesterol, triglycerides, and LDL-cholesterol and increased levels of HDL cholesterol [163].

In a double-blind, controlled trial of 62 obese subjects, subjects were randomly assigned to take five grams of either triphala (*n* = 31) or placebo (*n* = 31), two times daily for 12 weeks. No adverse effects or significant changes in liver and kidney function tests were observed in either group. Body weight, mean fasting blood sugar, and fasting serum insulin were all significantly decreased in the triphala group compared to placebo [164]. Larger double-blind, randomized controlled trials were carried out to test mouthwash formulations in people with periodontal diseases. Triphala extract mouth rinse was effective in reducing plaque accumulation and gingival inflammation, with no adverse effects [75,165,166].

Alzheimer’s disease is characterized by gut dysbiosis and activation of the innate immune system. Multiple pathogens have been identified in the oral cavities and brains of patients with Alzheimer’s, such as spirochetes, oral bacteria, herpes viruses, and fungi, which could trigger this innate immune response [15]. Therefore, treatment with triphala represents one of the strategies to reduce the chronic activation of the innate immune system in AD.

## 2. Other Medicinal Plants for AD

There are several other medicinal plants that have a role in the prevention or treatment of AD. However, in vitro or in vivo studies pertaining to their role in AD are very limited, the majority of the data are from observational studies, and there are no studies to support their role in preventing dementia. These plants include vacha (*Acorus calamus*), guduchi (*Tinospora cordifolia*), guggul (*Commiphora wightii*), jatamansi (*Nardostachys jatamansi*), jyotismati (*Celastrus paniculatus*), rosemary (*Rosmarinus officinalis*), Green tea (*Camellia sinensis*), St john’s wort (*Hypericum perforatum*), sage (*Salvia* spp), *Rhodiola rosea*, *Moringa oleifera*, shilajit, and lemon balm.

## 3. Administration of Herbs

The biggest challenge to drug delivery into the brain is circumventing the BBB, which prevents the entry of numerous potential therapeutic agents. While oral administration of the herbs is a common route of administration, there are no clear studies to demonstrate whether the herbal components have access to the CNS from the systemic circulation. Intranasal administration (INA) is non-invasive, rapid, bypasses the BBB, and directly targets the CNS [17,167,168,169,170,171]. Using this route of delivery, herbs in the form of dry powders or medicated oils are directly administered. Medicated oils may contain a mix of lipophilic and lipid-soluble molecules to ensure the synergistic interaction between different constituents in the herb. The benefits of INA include minimizing the side effects associated with systemic administration, avoidance of brain injury, and overcoming the need for implanting delivery devices [172]. Using this technique, researchers have treated memory losses in transgenic mouse models of AD [173]. While INA may be of great value, several contradictory findings in research studies limit its clinical value [173,174]. Though an attractive strategy in traditional medicinal systems for CNS conditions, there are not many clinical studies to support the use of INS for herbal delivery.

Another method of herbal administration involves the application of a medicated oil on the body and massaging the areas with gentle or deep hand movements. Massage reduces the levels of stress-related hormones and also triggers rapid cerebral blood flow [17,175,176,177,178]. Yet another mode of administration is a transcranial application of medicated oils so that the herbal extracts in the oil are in contact with the cranium or the frontal regions of the brain [17,179,180]. Recent studies point to the role of the endothelial cells lining the CNS capillaries in facilitating the entry of the solutes from the oil into the frontal lobe and prefrontal cortex [17,179,180,181].

## 4. Conclusions and Future Directions

An estimated 5.8 million Americans suffer from Alzheimer’s dementia. The number of patients with Alzheimer’s or other dementias may grow to a projected 13.8 million by 2050. In 2019, an estimated USD 290 billion was spent in the United States alone on healthcare expenses and lost wages for AD patients and their caregivers. The prediction is that by 2050, USD 1.1 trillion will be spent in the United States on AD [1,2]. Thus, there is an urgent need to find new therapeutics for the prevention and treatment of AD. While many new approaches to symptomatic treatment and disease-modifying therapy are currently under development for AD, the final outcome of their development to the market is uncertain [1,2,182,183]. A significant shift from a mono-therapeutic approach to a comprehensive, individualized, multi-therapeutic approach, especially for chronic and complex diseases such as AD, may be effective [13,14].

We have developed and implemented a comprehensive, precision medicine protocol for addressing AD-associated cognitive decline and documented sustained improvement in 100 patients [15]. The approach is personalized and targets suboptimal metabolic parameters. Optimizing these metabolic parameters has proven effective in several individuals with early AD or the pre-AD conditions, MCI or SCI.

One of the interventions that we use to normalize the metabolic parameters is medicinal herbs, which have a wide gamut of physiological actions that ultimately enhance memory and restore normal cognitive functions. Medicinal herbs have been the single most productive source of leads for the development of drugs, with over 100 new products in clinical development [184]. Herbs are more commonly prescribed in isolation, as a mix of several herbs, as is the case with triphala, or as an herbal extract. Administration of a single herb or a mixture of herbs allows for more efficacy, less non-specific toxicity, and, more importantly, it avoids drug resistance. Herbs have a long track record of safety and efficacy, which is most likely due to their multiple components and to the interactions of these different components with multiple physiological targets in the body [21,22,185]. The synergy between different constituents in the herb has been documented in several pharmacological activities [186]. Synergistic interactions include, among others, complementary mechanisms of action, such as immunomodulation, the reversal of resistance, and minimizing adverse effects. Thus, it is not surprising that a single herb or a mixture of herbs is preferred over an isolated compound or its derivative [21,22,185,187].

The evidence presented here highlights the largely unexplored potential of herbal medicines and adopting similar systems-based, individualized therapeutic approaches for AD. Further investigation into the biological mechanisms of the herbs, together with large multicenter clinical trials, is needed to validate the efficacy of both the single herbs and the mixed formulations in the treatment of Alzheimer’s disease and pre-Alzheimer’s cognitive compromise. More rigorous research is needed to overcome methodological limitations, including poor study design, relatively small sample sizes, poor outcome measures, and improper end-point selections [188,189]. It is hoped that the historical knowledge base of traditional systems of medicine, coupled with combinatorial sciences and high-throughput screening techniques, will improve the ease with which herbal products and formulations can be used in the drug development process to provide new functional leads for AD.

## Figures and Tables

**Table 1:** Neuroprotective herbs for the management of AD have a wide gamut of physiological actions. Listed below are the neurotherapeutic properties of these herbs that ultimately enhance memory and restore normal cognitive functions.

Herb	Study Type	Function/Outcome Measure	Reference
Ashwagandha (*Withania somnifera*)	in vitro, in vivo, clinical studies	antioxidant, anti-inflammatory, blocks Aβ production, inhibits neural cell death, dendrite extension, neurite outgrowth and restores synaptic function, neural regeneration, reverses mitochondrial dysfunction, improves auditory–verbal working memory, executive function, processing speed, and social cognition in patients	[20,23,24,25,26,27,28,29]
Brahmi (*Bacopa monnieri*)	in vitro, in vivo, clinical studies	antioxidant, anti-inflammatory, improves memory, attention, executive function, blocks Aβ production, inhibits neural cell death, delays brain aging, improves cardiac function	[30,31,32,33,34,35,36,37]
Cat’s claw (*Uncaria tomentosa*)	in vitro, in vivo, pre-clinical studies	anti-inflammatory, antioxidant, inhibits plaques and tangles, reduces gliosis, improves memory	[38,39,40,41,42,43,44,45]
Ginkgo biloba	in vitro, pre-clinical, clinical studies	antioxidant, improves mitochondrial function, stimulates cerebral blood flow, blocks neural cell death, stimulates neurogenesis	[46,47,48,49,50]
Gotu kola (*Centella asiatica)*	in vitro, in vivo, clinical studies	neuroceutical, cogniceutical, reduces oxidative stress, Aβ levels, and apoptosis, promotes dendritic growth and mitochondrial health, improves mood and memory	[51,52,53,54,55,56,57,58]
Lion’s mane (*Hericium erinaceus*)	in vitro, in vivo, pre-clinical and clinical studies	neuroprotective, improves cognition, anti-inflammatory, blocks Aβ production, stimulates neurotransmission and neurite outgrowth	[59,60,61,62,63]
Saffron (*Crocus sativus)*	in vitro, in vivo, clinical studies	antioxidant, anti-amyloidogenic, anti-inflammatory, antidepressant, immunomodulation, neuroprotection	[64,65,66]
Shankhpushpi (*Convolvulus pluricaulis*)	in vitro, in vivo, pre-clinical studies	promotes cognitive function, slows brain aging, antioxidant, anti-inflammatory	[33,36,67,68,69,70].
Triphala *(Emblica officinalis,* *Terminalia bellerica,* and *Terminalia chebula*)	in vitro, in vivo, pre-clinical and clinical studies	antioxidant, anti-inflammatory, immunomodulation, prevents dental caries, antibacterial, antiparasitic, reverses metabolic disturbances	[71,72,73,74,75,76].
Turmeric (*Curcuma* *longa*)	in vitro, in vivo, pre-clinical and clinical studies	antioxidant, anti-inflammatory, antimicrobial, blocks Aβ production, inhibits neural cell death	[77,78,79,80,81,82,83,84,85].

## Data Availability

Not Applicable.
